# Incidentally Detected Bilobed Testis in an Asymptomatic Adult Male: An Uncommon Variant of Polyorchidism

**DOI:** 10.1155/criu/9963636

**Published:** 2026-06-29

**Authors:** Rachel Cockburn, Jennifer Xu, Anthony Kiosoglous

**Affiliations:** ^1^ Faculty of Medicine, University of Queensland, Brisbane, Queensland, Australia, uq.edu.au; ^2^ Department of Urology, Queen Elizabeth II Jubilee Hospital, Brisbane, Queensland, Australia, qld.gov.au

## Abstract

Bilobed testis is an extremely rare congenital anomaly considered an incomplete form of polyorchidism, resulting from incomplete division of the genital ridge during embryogenesis. Fewer than 250 cases of polyorchidism have been reported worldwide, and bilobed variants represent only a small proportion of these cases. Recognition of this benign anatomical variant is important to avoid unnecessary surgical intervention. We report the case of a 37‐year‐old asymptomatic male in whom a left scrotal mass was detected incidentally on clinical examination. Scrotal ultrasonography demonstrated a well‐defined ovoid nodule adjacent to the posterior–inferior aspect of the left testis measuring 14 × 15 × 8 mm. The lesion demonstrated identical echogenicity and internal architecture to the adjacent testicular parenchyma, consistent with a bilobed testis. Follow‐up ultrasonography at 18 months showed no interval change, confirming stability. Given the benign imaging features, absence of symptoms and stability on serial imaging, the patient was managed conservatively with reassurance and education regarding testicular self‐examination. This case highlights the importance of recognising bilobed testis as a rare but benign congenital variant. Accurate radiological diagnosis can prevent unnecessary surgical exploration, reduce patient anxiety and preserve fertility.

## 1. Introduction

Polyorchidism is a rare congenital anomaly defined by the presence of more than two testes, with fewer than 250 cases reported in the literature [1]. The most common form is triorchidism (three separate testes), whereas incomplete variants such as bilobed testis are exceptionally uncommon. Bilobed testis is considered an incomplete manifestation of polyorchidism resulting from partial division of the genital ridge during early embryologic development.

Most cases are discovered incidentally during imaging for unrelated scrotal pathology. High‐resolution ultrasonography plays a key role in diagnosis, typically demonstrating a well‐defined structure with echotexture and vascularity identical to normal testicular parenchyma. Recognition of this variant is important, as it may mimic a testicular mass on imaging.

We report a case of incidentally detected bilobed testis in an adult male managed conservatively with imaging surveillance.

## 2. Case Report

A 37‐year‐old, otherwise well, male was referred for scrotal imaging following incidental detection of a palpable left scrotal mass on clinical examination. He reported no scrotal pain, swelling, trauma, infection or lower urinary tract symptoms. He had completed his family with two children. There was no history of cryptorchidism or family history of testicular cancer.

**Figure 1:**
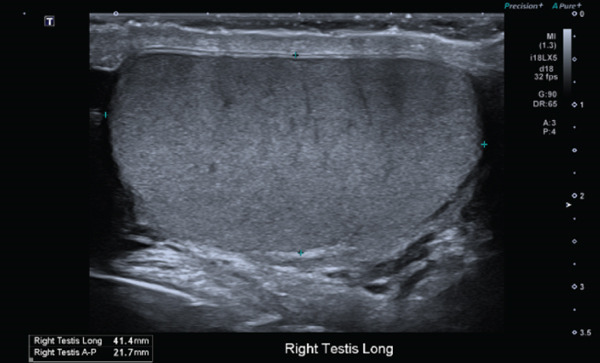
The right testicle on ultrasonography measures 41 × 22 × 29 mm, 14 cc and appears sonographically normal.

**Figure 2:**
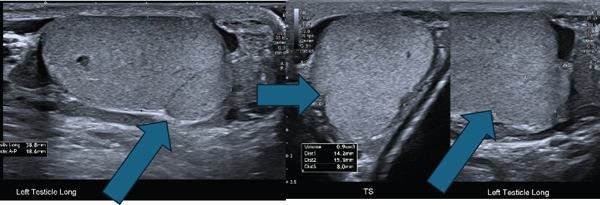
The left testicle measures 35 × 19 × 30 mm, 12 cc. On ultrasonography, it demonstrates a well‐defined, ovoid and solid nodule measuring 14 × 15 × 8 mm with identical echogenicity and internal architecture to the adjacent testicular parenchyma. Arrows demonstrate the ridge between each lobe of the left testicle.

Initial scrotal ultrasound demonstrated normal right and left testes with preserved parenchymal echotexture and vascularity. The right testis measured 13 cc in volume, with a small 4‐mm epididymal head cyst (Figure 1). The left testis measured 12 cc in size and had a normal appearance; however, immediately adjacent to its posterior–inferior aspect was a well‐defined, ovoid and solid nodule measuring 14 × 15 × 8 mm (Figure 2). The lesion demonstrated identical echogenicity and internal architecture to the adjacent testicular parenchyma. No hydrocoele, varicocoele, inflammatory change or regional lymphadenopathy was identified.

**Figure 3:**
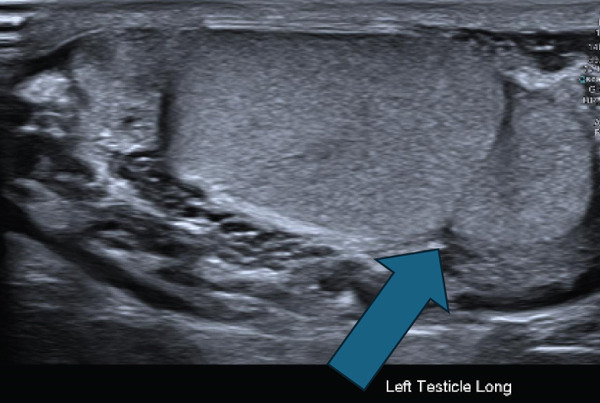
A follow‐up ultrasound performed 18 months later demonstrated no change, continuing to display identical echotexture, supporting the diagnosis of a bilobed testis. Arrow demonstrating ridge between each lobe of the left testicle.

The radiological appearance was suggestive of a bilobed testis, representing incomplete unilateral polyorchidism. A follow‐up ultrasound performed 18 months later demonstrated no change, continuing to display identical echotexture, supporting the diagnosis of a bilobed testis (Figure 3).

Given the absence of symptoms, stability on serial imaging and lack of concerning radiological features, this patient was managed conservatively with ultrasound follow‐up every 2 years. The patient was reassured regarding regular testicular self‐examination and discharged to their primary care provider for ongoing follow‐up.

## 3. Discussion

Polyorchidism is a rare congenital condition, with fewer than 250 cases reported worldwide [1]. Triorchidism is the most common form [2], whereas incomplete variants such as bilobed testis are exceptionally uncommon [3]. Bilobed testis is considered an incomplete expression of polyorchidism resulting from longitudinal or transverse division of the genital ridge between the fourth and eighth weeks of gestation [4]. Complete division produces a supernumerary testis, whereas incomplete division results in a bilobed configuration with shared parenchyma or ductal structures [5].

A review of the contemporary literature identified only one published case of bilobed testis within the last 5 years [6], highlighting the exceptional rarity of this condition. Haffar et al. reported a 39‐year‐old male presenting with a left‐sided testicular mass, in whom ultrasonography demonstrated a bilobed testis with normal vascularity and echogenicity identical to the adjacent testicular parenchyma. Similar to our patient, the lesion was initially detected as a palpable scrotal abnormality, demonstrated characteristic benign imaging features and was managed conservatively with surveillance and self‐examination.

Several classification systems have been proposed for polyorchidism. The traditional Leung classification categorises supernumerary testes according to their embryological relationship with the epididymis and vas deferens [7], whereas the more contemporary Bergholz classification groups cases according to reproductive potential and drainage through the vas deferens [8]. Bilobed testis does not fit neatly within these classifications because it represents incomplete duplication rather than a true supernumerary testis.

Most cases of polyorchidism are detected incidentally during childhood or adolescence [9, 10], either on imaging or during assessment for unrelated scrotal pathology. High‐resolution ultrasonography remains the diagnostic modality of choice [9], with homogeneous echotexture and preserved vascularity supporting a benign congenital variant and reliably excluding testicular neoplasia [11]. MRI may be useful in equivocal cases [12], demonstrating signal characteristics identical to normal testicular tissue and helping to exclude neoplastic lesions. Accurate radiological recognition is important because a bilobed testis may mimic a testicular mass and result in unnecessary surgical exploration.

The principal clinical concerns relate to malignancy and torsion. A large systematic review demonstrated that approximately 76% of supernumerary testes are scrotal, with malignancy occurring predominantly in extrascrotal, undescended or dysgenetic gonads [9]. Reported malignancy rates in polyorchidism range from 1% to 7% [8], although no cases of malignant transformation arising from a bilobed testis have been conclusively documented [12]. Bilobed testes account for approximately 10% of reported supernumerary variants, and no cases of malignancy arising from a bilobed testis have been confirmed [13].

Although torsion has been described in polyorchidism [14], the absolute risk remains low, and most uncomplicated intrascrotal cases are now managed conservatively. Historically, surgical exploration and excision were frequently performed because of uncertainty regarding malignant potential [8]. Contemporary literature supports observation with clinical and ultrasonographic surveillance in asymptomatic patients with benign imaging features [13, 15, 16]. Some authors have suggested annual clinical examination and ultrasonography, although no evidence‐based surveillance protocol currently exists [9].

This case reinforces bilobed testis as a rare but benign anatomical variant. Accurate radiological recognition can prevent unnecessary surgical exploration, reduce patient anxiety and avoid iatrogenic compromise to fertility.

## Author Contributions

**Rachel Cockburn:** conceptualisation, investigation, writing—original draft and writing—review and editing. **Jennifer Xu:** conceptualisation and writing—review and editing. **Anthony Kiosoglous:** supervision and writing—review and editing.

## Funding

Open access publishing facilitated by The University of Queensland, as part of the Wiley ‐ The University of Queensland agreement via the Council of Australasian University Librarians.

## Disclosure

All authors have read and approved the final version of the manuscript. Rachel Cockburn had full access to all of the data in this study and takes complete responsibility for the integrity of the data and the accuracy of the data analysis.

## Consent

Written informed consent has been obtained from the patient to publish this paper.

## Conflicts of Interest

The authors declare no conflicts of interest.

## Data Availability

The data that support the findings of this study are available on request from the corresponding author. The data are not publicly available due to privacy or ethical restrictions.
